# Crescentic glomerulonephritis associated with syphilis: a case report and review of the literature

**DOI:** 10.1186/s13256-023-04293-2

**Published:** 2023-12-22

**Authors:** Akiko Kaiga, Yuka Sato, Haruna Arakawa, Tatemitsu Rai, Akihiro Tojo

**Affiliations:** 1 Department of Nephrology & Hypertension, Dokkyo Medical University, 880 Kitakobayashi, Mubu, Tochigi Japan; 2 Department of Nephrology & Hypertension/Blood Purification Center, Dokkyo Medical University Hospital, 880 Kitakobayashi, Mubu, Tochigi 321-0293 Japan

**Keywords:** Syphilis, Crescentic glomerulonephritis, Rapidly progressive glomerulonephritis, Electron microscopy

## Abstract

**Background:**

Crescentic glomerulonephritis with syphilis infection is rare, and the mechanism underlying the formation of glomerular capillary wall damage-induced crescent has not been elucidated.

**Case presentation:**

A 62-year-old Japanese male showed edema, eruption, and rapid deterioration of the renal function after an acute syphilis infection. A renal biopsy showed crescentic glomerulonephritis with C3 deposition in the glomerular capillary wall, and immunostaining for anti-*Treponema pallidum* antibody was weakly positive in some interstitium and one glomerulus. Electron microscopy revealed the presence of string-shaped structures in the glomerular capillary walls. After treatment with penicillin followed by prednisolone, the renal function and urinary abnormalities, including *Treponema pallidum* protein, disappeared.

**Conclusions:**

Crescentic glomerulonephritis associated with syphilis showed a string-shaped deposition in the glomerular capillary and urinary *Treponema pallidum* protein excretion, and was effectively treated with penicillin and prednisolone.

## Background

Membranous nephropathy is well known as a renal disease associated with syphilis [1–4]. Antibodies against *Treponema pallidum* (TP) may form immune complexes that deposit in the subepithelial regions of the glomerular wall, causing secondary membranous nephropathy [2]. In fact, antibody elution studies performed on the renal biopsy specimen demonstrated the presence of anti-treponemal antibody within the glomerular immune complex deposits [2]. However, there is also a report that renal tissue damage occurs due to the direct infiltration of TP into renal tissue.

Recently, cases of pauci-immune crescent nephritis with rapidly progressive glomerulonephritis (RPGN) have been reported in chronic syphilis infection with positive TP particle reaction (TPPA) but negative rapid plasma regain (RPR) [5] and active infection with positive reactions for both RPR and TPPA [6].

We herein report a case of crescentic glomerulonephritis after acute syphilis infection with string-shaped structures in the basement membrane, as observed by electron microscopy.

## Case presentation

A 62-year-old Japanese man visited our hospital complaining of edema in the legs and hands. Two months ago, he had had sexual intercourse with a professional sex worker, after which he had experienced eruption and itching in his inner thighs and lower legs. He was being treated with angiotensin receptor blockers and calcium channel blockers, and his hyperuricemia was being treated with selective urate reabsorption inhibitor and xanthine oxidase inhibitor, and hypercholesterolemia with statin; however, the results of urinalysis were almost normal.

One week before he recognized edema, his body weight had increased 5 kg from the usual body weight. A physical examination revealed a body temperature of 36.4 ℃, blood pressure of 177/85 mmHg, pulse rate of 65/min, SpO_2_ of 99% (room air), and normal heart and lung sounds with dilated abdomen and legs edema. The eruption in his inner thighs and lower legs had disappeared, and no inguinal lymph node swelling was observed.

Laboratory data showed that proteinuria at 5.87 g/day with a selectivity Index of 0.305. Urinary sediments showed many red blood cells (RBCs)/high power field (HPF), 5–9 white blood cells (WBCs)/HPF, 1–4 hyaline casts/HPF, a few granular casts/whole field (WF), 1–3 lipid casts/low power field (LPF), and a few RBC casts/WF. A blood examination showed 9600 × 10^4^ WBCs/μL, Hb 13.1 g/dl, 29.3 × 10^4^ platelets/μL, serum albumin 2.9 g/dl, total protein 6.9 g/dL, urea nitrogen 23.5 mg/dL, creatinine (Cr) 1.33 mg/dL, estimated glomerular filtration rate (eGFR) 43.2 ml/min/1.73m^2^, C-reactive protein (CRP) 0.94 mg/dL, immunoglobulin G (IgG) 1469 mg/dL, IgA 416 mg/dL, IgM 54 mg/dL, C_3_ 170 mg/dL, C_4_ 44 mg/dL, myeloperoxidase (MPO)-antineutrophil cytoplasmic antibody (ANCA) < 1.0 U/mL, proteinase 3 (PR3)-ANCA < 1.0 U/mL, anti-glomerular basement membrane (GBM) antibody 2.0 U/mL, rheumatoid factor 3.8 IU/mL, antinuclear antibody 20x, anti-streptolysin O antibody 131 IU/mL, RPR test positivity 20.5x, TP hemagglutination (TPHA) test positivity, hepatitis B surface (HBs) antigen < 0.05 IU/mL, ant-hepatitis C virus (HCV) antibody negativity, and human immunodeficiency virus (HIV) negativity. He was diagnosed with acute glomerulonephritis and nephrotic syndrome and admitted to our hospital for renal biopsy and treatment.

**Fig. 1:**
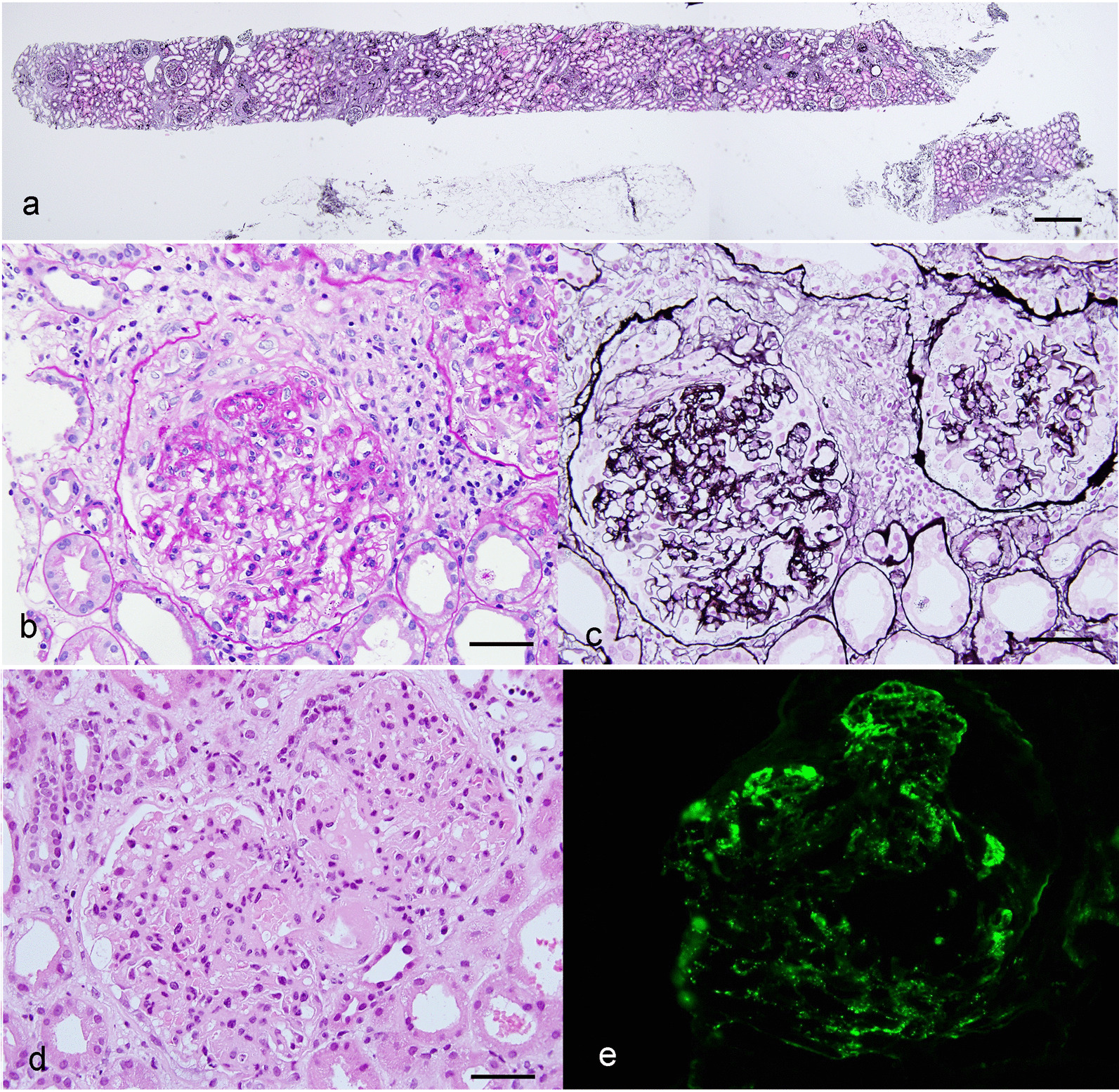
Light microscopy of the renal biopsy samples. PAM staining (**a**, **c**), PAS staining (**b**), HE staining (**d**), and immunofluorescence for C3 staining (**e**). The bars indicate 400 µm (**a**) and 50 µm (**b**–**d**)

**Fig. 2:**
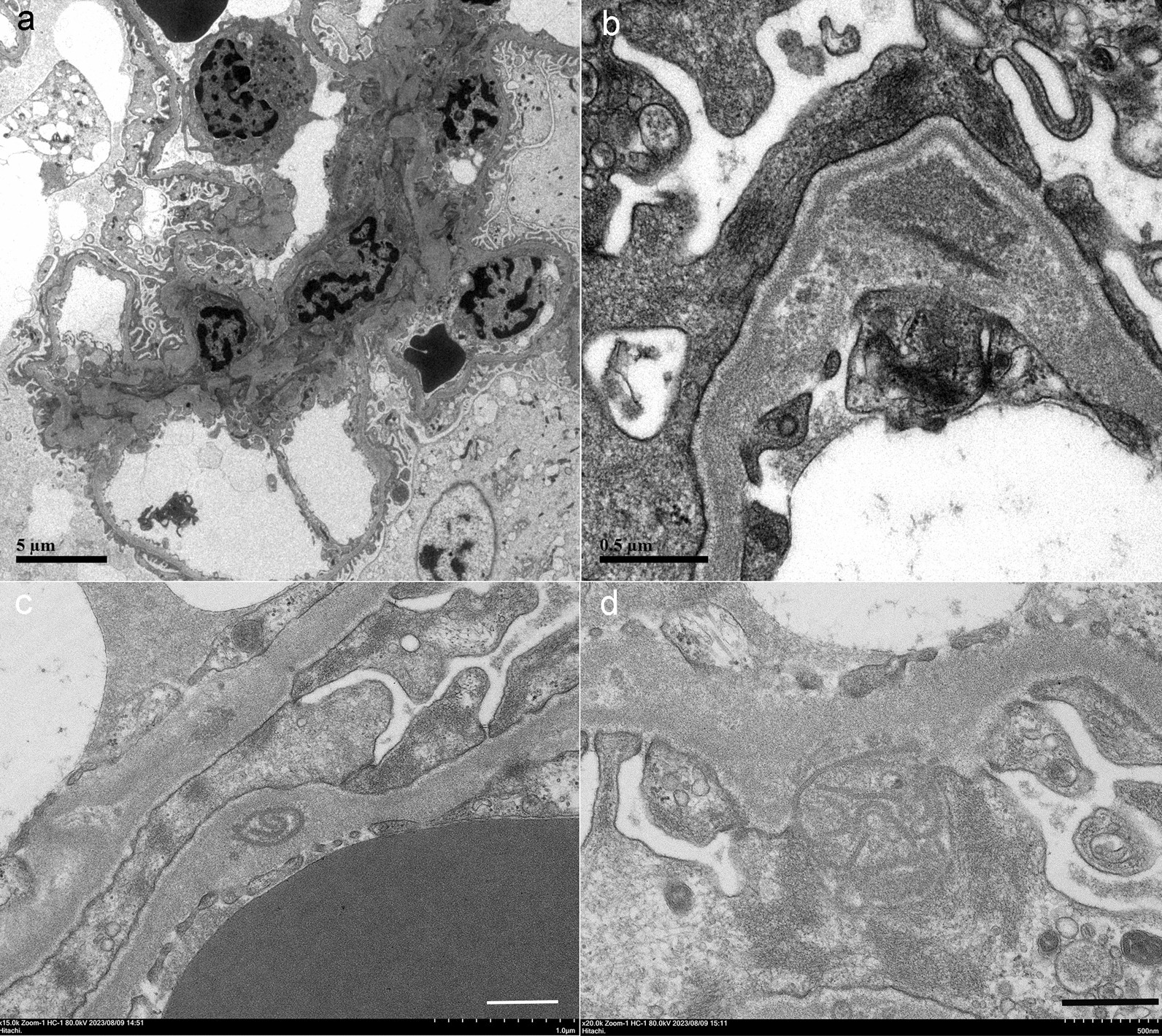
Electron microscopy of the renal biopsy samples. No electron-dense deposits were noted along the capillary walls (**a**), but string-like structures with granular particles were noted in the subendothelial space (**b**), glomerular basement membrane (**c**), and subepithelial area (**d**)

The renal biopsy showed 24 glomeruli, including 4 with global sclerosis (17%), 5 with crescents (21%), and focal tubular atrophy and fibrosis (Fig. 1a). The presence of fibrocellular crescents in 5 glomeruli (Fig. 1b, c) with fibrinoid necrosis in one, and mesangial and endocapillary proliferation in 9 glomeruli with intraglomerular neutrophil infiltration (Fig. 1d). Immunofluorescence of C3 immunofluorescence was positive along the capillary wall (Fig. 1e), but immunofluorescence of IgA, IgG, IgM, kappa, lambda, C1q and C4 were negative. These findings in the light microscopy and immunofluorescence were consistent with the diagnosis of post-infectious glomerulonephritis. Electron microscopy showed no electron-dense deposits in the subepithelial area, excluding a diagnosis of membranous nephropathy (Fig. 2a). Interestingly, a string-like structure was observed in the subendothelial space, GBM, and subepithelial area (Fig. 2b–d).

**Fig. 3:**
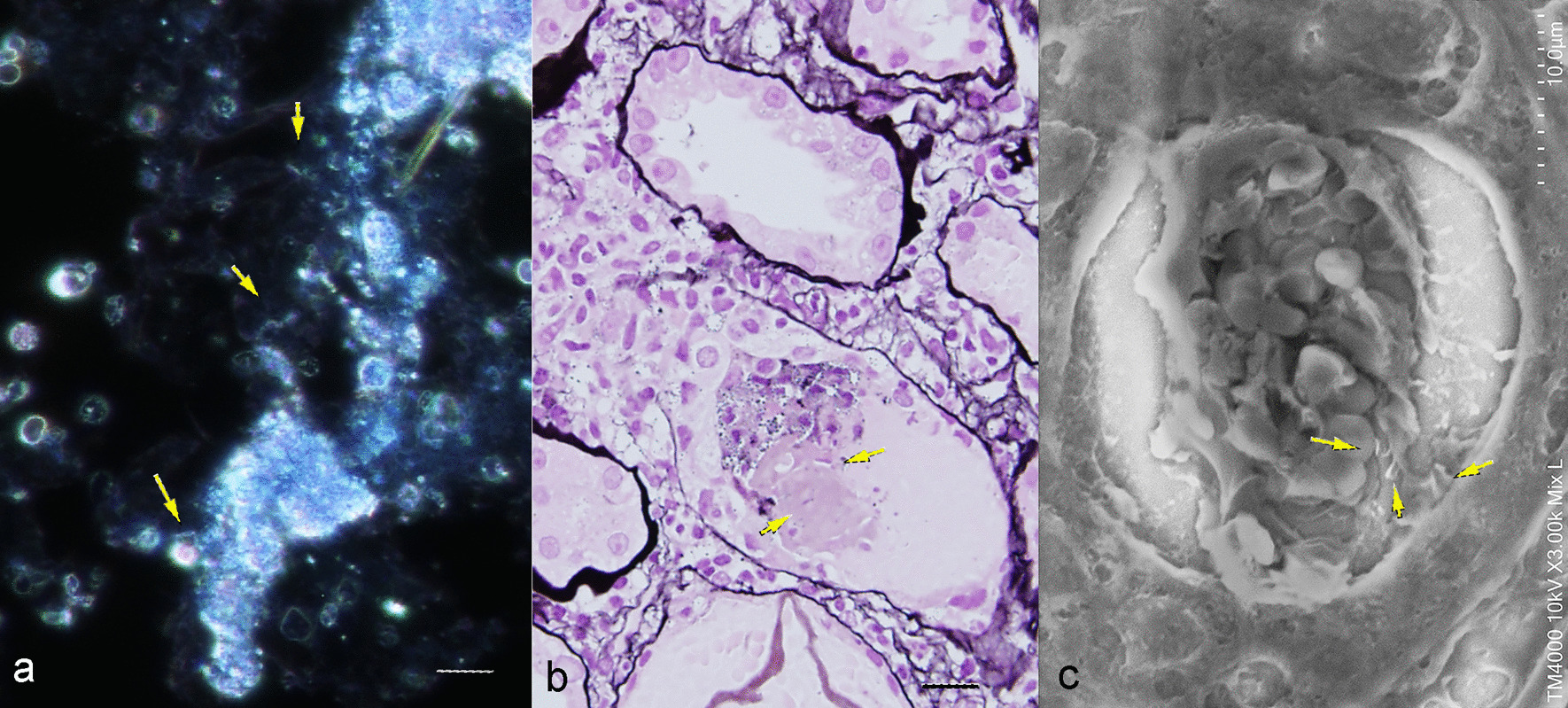
Urinary sediments with dark-field observation (**a**), PAM staining of the renal biopsy sample, and an LVSEM image of the renal biopsy sample (**c**). The arrows indicate rod bacteria. The bar indicates 20 μm in an and **b**

**Fig. 4:**
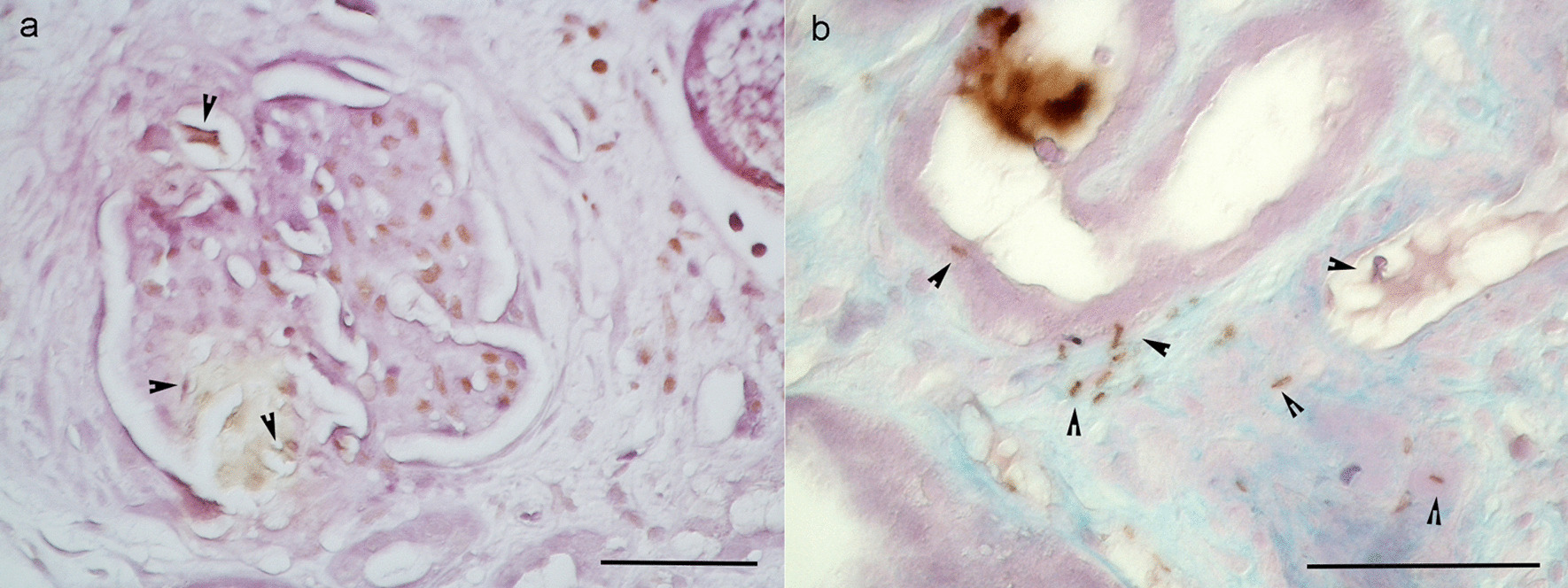
Immunohistochemistry against rabbit polyclonal anti-*Treponema pallidum* antibody (Biocare Medical, Pacheco, CA, USA, 1:100 dilution) in glomerulus (**a**) and tubulointerstitial region (**b**). Arrowheads indicate positively stained bacteria, suggesting *Treponema pallidum*. The bar indicates 50 μm

**Fig. 5:**
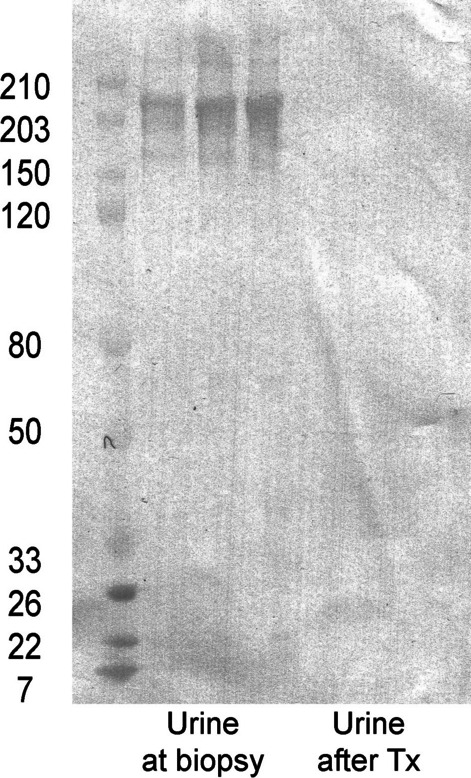
Western blotting for anti-*Treponema pallidum* antibody (1:500 dilution) in the urine at the renal biopsy and in the urine after treatment with antibiotics

A urinalysis showed WBC casts with glittering bacteria by dark-field microscopy (Fig. 3a), attached to tubular casts by Periodic acid-methenamine silver (PAM) and low vacuum scanning electron microscope (LVSEM) observation (Fig. 3b, c). Immunohistochemistry for anti-TP antibody was negative in most glomeruli but weakly positive in some interstitium, some casts, and one glomerulus (Fig. 4). Western blotting revealed that the urinary protein at the renal biopsy showed a band for TP that disappeared after antibiotic treatment (Fig. 5).

**Fig. 6:**
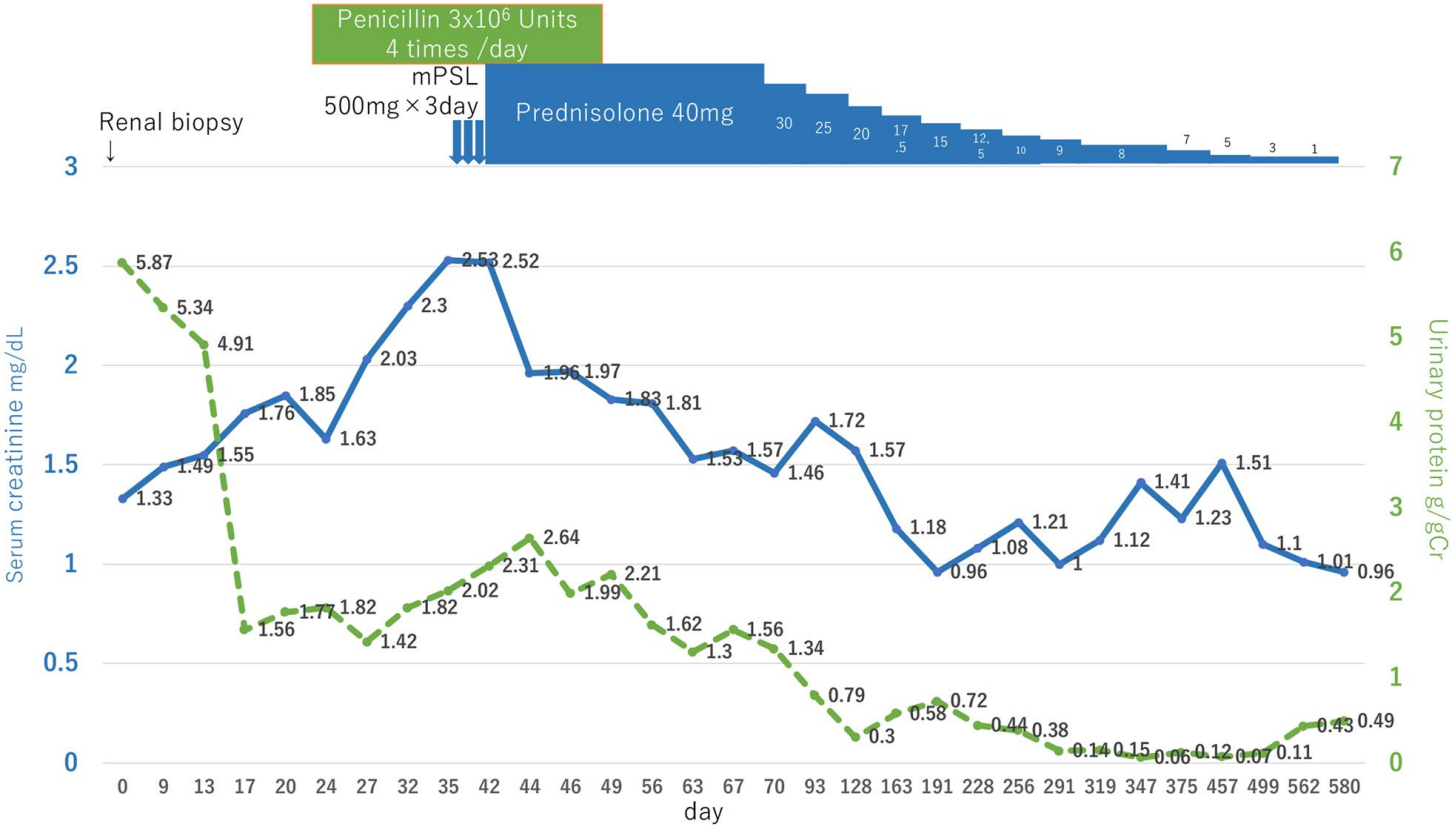
Changes in proteinuria and serum creatinine levels after treatment with penicillin and prednisolone

After treatment for TP by penicillin (12 × 10^6^ U/day) for 2 weeks, the serum creatinine levels had not improved. A renal biopsy diagnosed post-infectious crescentic glomerulonephritis, so methylprednisolone pulse therapy (mPSL) was applied, followed by oral steroids at 40 mg. The serum creatine level finally turned to 0.96 mg/dl at prednisolone 1 mg with proteinuria of 0.49 g/gCr after 580 days (Fig. 6).

## Discussion

Renal disease due to syphilis infection is uncommon but is observed in 0.3% of secondary syphilis infections [7]. In the first stage of syphilis, after a three-week latent period of TP mucosal infection, the initial induration appears locally, forming a hard chancre. This spontaneously disappeared, and swelling of the regional lymph nodes and indolent bubo appears, lasting for approximately nine weeks. In the second stage of syphilis, TP spreads through the bloodstream and metastasizes to various organs of the body, causing skin rashes, generalized condylomata, and lesions of bones, joints, and eyes. This stage lasts from one to three years, with repeated relapses. Nephropathy also develops during the second stage of the infection.

Renal manifestation is commonly membranous nephropathy, caused by the deposition of treponemal antigen-antitreponemal antibody complexes. Antibiotic therapy for syphilis improves renal diseases [1, 2]. Antibody elusion study of the renal biopsy specimen revealed the presence of anti-treponemal antibody within the subepithelial immune deposits of syphilis-associated membranous nephropathy [2]. PLA2R antigen was positive in 40% with membranous nephropathy associated with syphilis [8]. In addition, neuron-derived neurotrophic factor (NDNF) was identified as a novel antigen in all five cases of syphilis-associated membranous nephropathy by mass spectrometry [9].

In addition to membranous nephropathy, there are several case reports of crescentic glomerulonephritis. TP is a weakly anaerobic Gram-negative bacterium belonging to the spirochete family. It is a spiral bacterium with a diameter of 0.1–0.4 μm and length of 6–20 μm [10]. A bundle of 6–8 intracytoplasmic microtubules with a diameter of 7 nm runs longitudinally in the cell body. It has been reported that the anti-TP antibody is positive in the glomerulus and may cause rapidly progressive glomerulonephritis owing to direct renal infiltration [11]. In our case, a renal biopsy was performed before antibiotic treatment, and most glomeruli were negative for anti-TP antibodies, but weak staining was observed in some interstitium, some casts, and one glomerulus (Fig. 4). Polarizing microscope images of the urinary sediment showed 2-to 10-μm TP-sized bacteria around the leukocyte casts, renal biopsy PAM staining showed TP-like bacteria had adhered to renal tubular casts, and LVSEM images showed TP-like bacteria around the casts (Fig. 3). Electron microscopy revealed string-like structures within the glomerular subendothelial space, GBM, and subepithelial space, which were smaller in size than the TP bacteria (Fig. 2). However, it is likely that these structures activate C3 and disrupt the GBM to form a crescent. These string-like structures may be part of the structures, such as microtubules within spirochetes. Electrophoresis of the patient’s urine at the renal biopsy showed a protein with a molecular weight of 206 kDa that stained for anti-TP antibody but was absent in the urine sample examined after antibiotic treatment. (Fig. 5), suggesting that some components of TP are excreted in urine during the active phase and activate C3 to cause tissue damage. Recently, RPGN with syphilis presenting with crescentic glomerulonephritis was reported and was successfully treated with penicillin and early steroid therapy, as shown in Table 1 [5, 6, 11]. The present patient also achieved complete remission with penicillin and steroids (Fig. 6). Our case is important, as it proposes a new perspective for considering the mechanism underlying crescent formation.

**Table 1:** Rapidly progressive glomerulonephritis associated with syphilis

	Age (years), sex	Symptoms	Syphilis test RPR, TPHA	BP (mmHg)	Urinary protein, sediment	ANCA GBM-Ab	Serum creatinine (mg/dL)	Pathology	Treatment	Hemodialysis		Outcomes
Walker, Am J Med, 1984	37, male	No skin lesion, or penile lesion, dark urine, oliguria, edema	VDRL 1:512, TP-Ab 1:16	180/115	UP 4 + , RBC 100/HPF	ND	21.6	Cellular crescent > 90% glomeruli	mPSL pulse Plasmapheresis 3 times/week benzathine penicillin 2.4 M units/week for 4 weeks	Short-term HD		Cr 1.5–1.8, NS VDRL 1:2
Nandikanti, Clin Nephrol, 2020	28, male	HIV + , AKI, NS, no skin lesion, no lymphadenopathy	1:32, +	146/78	4.1 g/gCr, RBC25-30/HPF. WBC 15–25/HPF	Negative	7.2	Necrotizing cellular crescent (6/9), segmental sclerosis (3/9), TI IF: C3 + , IgM + , EM: FPE, EDD	benzathine penicillin 2.4 M units/week for 3 weeks	(−)		Cr 1.5, UP 1.2 g/gCr, normal BP RPR 1:4
Qi, BMC Nephrol 2021	77, female	General fatigue, polyneuropathy, anasarca, no skin lesion, AKI	-/ + , VDRL +	ND	1.22 g/gCr, dysmorphic erythrocytes, eosinophils	Negative	2.61	Necrotizing crescent (9/23), TI, IF: mes C3, IgG, IgG4 in plasma cells, EM: no EDD	IV penicillin G 2 weeks mPSL 500 mg × 3 days	(−)	Cr 1.51 (1 week), Cr 1.24 (1 month), Cr 1.10 (1 year) VDRL ND	
Present case	62, male	Leg edema, RPGN, disappearance of skin eruption	1:20.5, +	177/85	5.87 g/gCr, many RBCs, 5–9 WBCs/HPF	Negative	2.52	Cellular crescent (5/24), Fibrinoid necrosis (1/24), TI	penicillin G 12 M/day 2 weeks, mPSL 500 mg × 3 days	(−)		Cr 0.96, UP 0.49 g/gCr RPR1:1.2

BP: blood pressure, UP: urinary protein, HD: hemodialysis, NS: nephrotic syndrome, RPR: rapid plasma reagin, TPPA: *Treponema pallidum* particle agglutination, VDRL: venereal disease research laboratory, ND: not described, IV: intravenous, M: million, EM: electron microscopy, FPE: foot process effacement, EDD: electron dense deposits, TI: tubulointerstitial lesion, mes: mesangial

## Conclusion

A case of crescentic glomerulonephritis with rapidly progressive glomerulonephritis after syphilis infection showed string-like structures in the glomerular capillary and urinary *Treponema pallidum* protein excretion, which may have played a pathological role in the formation of the observed crescent. Treatment with penicillin and steroids improved the renal function and proteinuria.

## Data Availability

Photographs, including light microscopy, immunofluorescence, electron microscopy, immunostaining, LVSEM, and Western blot, are shown as original data. Unlabeled photographs are available upon request to the corresponding author.
